# Effect of Online Product Presentation on the Purchase Intention of Wearable Devices: The Role of Mental Imagery and Individualism–Collectivism

**DOI:** 10.3389/fpsyg.2020.00056

**Published:** 2020-02-20

**Authors:** Jinnan Wu, Fang Wang, Lin Liu, Donghee Shin

**Affiliations:** ^1^School of Business, Anhui University of Technology, Ma’anshan, China; ^2^School of Management Science and Engineering, Anhui University of Technology, Ma’anshan, China; ^3^College of Communication and Media Sciences, Zayed University, Abu Dhabi, United Arab Emirates

**Keywords:** online product presentation, mental imagery, perceived social risk, positive emotion, wearable device, individualism–collectivism

## Abstract

The main objective of this study was to investigate how and when online product presentation influences individual purchase intention of wearable devices in China. This study hypothesized that online product presentation would influence individual mental imagery, which, in turn, would impact purchase intention through perceived social risk and positive emotion, but this effect would be moderated by individualism–collectivism value at the individual level. We performed a laboratory experiment (2 × 2) to collect the data (*N* = 254). The experimental results indicated that there was a significant interaction effect between the product feature presentation video and usage scenario presentation picture on mental imagery. In addition to a direct effect, mental imagery had an indirect effect on purchase intention through perceived social risk and positive emotion. Additionally, the behavioral effect of perceived social risk was moderated by individualism–collectivism. Specifically, compared with individualists, perceived social risk had a stronger impact on collectivists’ purchase intention. This study contributes to a greater understanding of the mechanism through which online product presentation drives purchase intention, with a particular emphasis on how individualism–collectivism value at the individual level moderates consumers’ intention to purchase wearable devices.

## Introduction

Wearable devices have recently attracted market attention (Shin and Biocca, 2018), yet they are still at an early stage of market diffusion and have not yet become mainstream (Nascimento et al., 2018). Given that most consumers purchase wearable devices via online channels, a further understanding of how to drive consumers to purchase wearable devices online is critical for both researchers and practitioners (Jeong et al., 2017; Hsiao and Chen, 2018; Lee and Lee, 2018).

The use of mental imagery or processing fluency has been well established in consumer behavior research and leveraged by marketers to influence consumers’ perceptions and choices (MacInnis and Price, 1987; Petrova and Cialdini, 2005; Lee and Gretzel, 2012; Wu K. et al., 2016). Hence, in online shopping settings, which lack a physical product experience, it is important to present vivid and rich information about product features and/or consumption to help consumers generate mental imagery (Lee and Gretzel, 2012; Wu K. et al., 2016; Flavián et al., 2017), which can compensate for the missing sensory experience, thus promoting consumers’ online purchase behavior (Park et al., 2005; Algharabat et al., 2017; Orús et al., 2017). Unlike extensive literature on the effect of online product presentation (e.g., videos, pictures, and text) on evoking mental imagery, this study aims to examine the interaction effect of different presentation stimuli. We aim to extend prior mental imagery processing research focusing on print advertising and usually including one external advertising stimulus (Babin and Burns, 1997; Lee and Gretzel, 2012).

Further, this study focuses on the mechanism through which mental imagery influences the purchase intention toward wearable devices. In line with prior findings that consumers’ cognitive and emotional responses are largely determined by online product presentation (Park et al., 2005; Müllerstewens et al., 2017; Orús et al., 2017), we argue that when consumers consider purchasing wearable devices, imagery processing can promote their purchase intention through two parallel psychological processes. One process motivates consumers’ purchase behavior by decreasing their perceived social risk. The other process changes consumers’ purchase behavior by stimulating their positive emotional responses through experiencing an immediate gratification of consumption situation and/or activating visual mental representation of emotional events (MacInnis and Price, 1987; Yoo and Kim, 2014). As a cutting-edge technology product in the early stage of market diffusion (Chuah et al., 2016; Yang et al., 2016), wearable devices have not been widely adopted and utilized by online consumers (Jeong et al., 2017). Thus, successful diffusion of wearable devices or technologies largely depends on stimulating consumers’ positive emotional responses and reducing their perceived social risk (Yang et al., 2016; Shang et al., 2017). Thus, another purpose of this study is to test whether mental imagery increases consumers’ purchase intention via decreasing perceived social risk and eliciting positive emotions, which will reveal the psychological process of new product adoption or purchase behaviors.

Technology adoption literature has suggested that a user’s initial adoption of an IT/IS innovation (e.g., wearable technology or device in this study) is determined by his/her attitude and perception of usefulness and ease of use (Davis et al., 1989; Karahanna et al., 1999), perceived risk (Pavlou, 2003), and emotional response (Venkatesh, 2000). However, very little research has been conducted across consumers holding different value orientations. The cultural influence of individualism–collectivism values at individual levels in technology adoption and product choice has recently been raised (Faqih and Jaradat, 2015; Gupta et al., 2019), but limited attention has been paid to whether individualism–collectivism value orientation moderates the relationship between risk and emotion-related factors and individual behavioral intention. This study contributes to the technology adoption literature by investigating whether individualism–collectivism at the individual level moderates the relationship between perceived social risk and positive emotion and purchase intention in China.

## Conceptual Framework and Research Hypotheses

### Consumer Adoption and Purchase Behavior of Wearable Technology

Recent studies have found that consumer attitude toward smartwatches is the significant determinant of adoption and/or continuing intention (Choi and Kim, 2016; Wu L.-H. et al., 2016; Hsiao and Chen, 2018). Additionally, consumers’ attitudes toward smartwatches or wearable technologies are influenced by cognitive factors, including perceived self-expressiveness, perceived usefulness, and ease of use (Choi and Kim, 2016; Wu L.-H. et al., 2016), and emotional factors, such as enjoyment, entertainment, and emotional value (Choi and Kim, 2016; Wu L.-H. et al., 2016; Yang et al., 2016; Cho and Lee, 2017; Hsiao and Chen, 2018). Some researchers have also suggested that consumers’ intention was predicted by product characteristics such as smartwatch novelty, design aesthetics, visual attractiveness, functionality, compatibility, and demonstrability of results (Choi and Kim, 2016; Wu L.-H. et al., 2016; Yang et al., 2016; Jeong et al., 2017; Hsiao and Chen, 2018). Further, consumer’s personality traits have been found to influence smartwatch adoption and purchase. For instance, Choi and Kim (2016) confirmed that consumer innovativeness, need for uniqueness, and vanity influenced consumers attitude and behavioral intention toward smartwatches. Additionally, social influence (Wu L.-H. et al., 2016; Yang et al., 2016), the subjective norm (Wu L.-H. et al., 2016), and interpersonal influence (Lee and Lee, 2018) have been confirmed to affect consumers’ adoption and diffusion of smartwatches or wearable devices.

### Mental Imagery

Mental imagery is conceptualized as the process by which perceptual or sensory information is represented in the working memory (i.e., the form of mental images) that are used in the same way as perceptions of external stimuli (MacInnis and Price, 1987; Goossens, 2000). Burns et al. (1993) argued that individuals can experience mental imagery in different sensory representations of ideas, feelings, and memories; that this mental imagery can vary in terms of vividness and quantity; and that mental imagery enables individuals to experience a sensory stimulus in the absence of a true stimulus. Much research from advertising literature has shown that when consumers lack actual product experiences, their mental imagery processing will be aroused by indirect product experiences, such as when you see a picture of a car, you may imagine yourself actually driving the car (Babin and Burns, 1997; Overmars and Poels, 2015). MacInnis and Price (1987) argued that the effect of self-related imagery on intentions might be explained in two ways: by the concreteness of the imagined scenarios and/or by the greater emotionality of the imagery. Even without a real stimulus, mental imagery enables individuals to experience a sensory stimulus (Rodríguez-Ardura and Meseguer-Artola, 2015). As such, consumers’ mental imagery toward a product represented in their minds becomes critical information sources for their judgments (Yoo and Kim, 2014). Prior studies have found that product pictures presented on a website can arouse consumers’ mental imagery, which, in turn, helps them to comprehend the product smoothly and to further influence their behavioral intention (Lee and Gretzel, 2012; Overmars and Poels, 2015). Although previous research has focused on print advertising and has usually included one external advertising stimulus (Babin and Burns, 1997; Lee and Gretzel, 2012), little is known about the interaction effects of more different stimuli, specifically in the context of shopping websites. Moreover, recent studies have highlighted the role of mental imagery in online shopping (Schlosser, 2003; Gavilan et al., 2014; Flavián et al., 2017) however, the mechanism through which mental imagery influences purchase intention online is unexplored. This study seeks to contribute to the mental imagery literature by testing the interaction effect of different presentation stimuli on mental imagery and identifying the missing link between mental imagery and purchase intention in the online shopping context.

### Online Product Presentation and Mental Imagery

Different forms of product presentation, including text, static and dynamic pictures, 3D, video, etc., have been found to create different levels of presentation vividness, which, in turn, affect consumers’ attitude and purchase intention (Orús et al., 2017). This study aims to examine the interaction effect of two usually investigated presentation stimuli, i.e., the product feature presentation video and usage scenario pictures, in enhancing consumers’ mental imagery. Pictures are well-established predictors of imagery because visual information tends to be remembered better than verbal information (MacInnis and Price, 1987; Walters et al., 2007; Wu K. et al., 2016). With the increased focus on the impact of product pictures in consumer imagery processing, understanding the conditions under which pictures produce imagery becomes important (MacInnis and Price, 1987). This study fills the gap by examining when product usage scenario pictures have a greater or weaker impact on mental imagery.

Recent research has discovered that the perceived ease of imagining the product influences the effectiveness of vivid information, such as the product presentation video (Flavián et al., 2017). A large amount of information leads to low processing fluency because people require more effort to extract physical features (Wu K. et al., 2016). Instead, the dynamic online product presentation can provide richer and more specific clues for activating consumer mental imagery than the static online production presentation (Overmars and Poels, 2015). Thus, when consumers are exposed to the presentation video, they will pay more attention to this dynamic and vivid visual information and subsequently evoke mental imagery of the product. At this time, limited cognitive resources will be allocated to process usage scenario information, even if usage scenarios are shown with concrete pictures (Petrova and Cialdini, 2005). Thus, by creating a cognitive load, video information about product features may occupy necessary resources and consequently undermine the effects of usage scenario pictures. Specifically, we expected that under product features dynamically presented with a video, the effect of usage scenario pictures was not ineffective in eliciting positive mental imagery.

**Hypothesis 1:** Product usage scenario pictures and feature textual information interact to increase mental imagery. When consumers are exposed to the product feature video, the pictures will result in weaker mental imagery. When they are exposed to product features with textual information, pictures will lead to stronger mental imagery.

### Mental Imagery Affecting Perceived Social Risk, Positive Emotion, and Purchase Intention

Consumers’ perception of social risk, the anticipated or potential lack of acceptance from significant others as a result of adopting a product or service (Featherman and Pavlou, 2003; San Martín et al., 2011), is increasingly recognized as one of the fundamental drivers of purchase decisions (Yokoyama et al., 2014; Shang et al., 2017). However, in the wearable products consumption context, much of the research up to now has confirmed the effect of performance risk and financial risk (Yang et al., 2016) as well as privacy risk (Gu et al., 2016) on user acceptance intention. It is unclear whether the relationship between perceived social risk and purchase intention still holds true for wearable devices.

Multisensory information such as vision, touch, and smell can be processed and integrated by mental imagery, which arouses consumers’ memories and imagination and has positive effects on their attitudes. The marketing literature has shown that mental imagery processing induced by an external marketing stimulus influences consumer cognitive and emotional responses (Babin and Burns, 1997; Yoo and Kim, 2014; Overmars and Poels, 2015). Since most people around us have no or little experience with smartwatches, consumers will perceive social risk when they plan to purchase smartwatches. This is a typical cognitive response affected by mental imagery. A recent study by Sobkow et al. (2016) indicated that visual mental imagery is associated with the degree of perceived risk. They argued that negative mental imagery leads to negative emotions and pressures, which, in turn, increase the perceived risk and ultimately reduce individuals’ willingness to engage in risk-taking behavior (Sobkow et al., 2016). In line with these findings, this research proposes that consumers’ mental imagery will be negatively associated with perceived social risk in the context of online shopping for smartwatches. Therefore, the following hypothesis is formulated:

**Hypothesis 2:** Mental imagery will be negatively related to the perceived social risk of purchasing smartwatches.

In addition to the effects on consumers’ cognitive responses, mental imagery also acts on their emotional responses (Yoo and Kim, 2014; Overmars and Poels, 2015). In accordance with the affect heuristics (Slovic et al., 2002; Slovic et al., 2007), when consumers make product judgments and purchasing decisions, a rich and vivid product presentation would automatically activate corresponding imagery and related emotions, triggering emotional responses to a product. This phenomenon has been empirically supported by Yoo and Kim (2014). Although negative emotion has been proven to affect consumers’ behavioral intention (Babin et al., 2013; Ki et al., 2017), positive emotional reactions are the most commonly used to explain user adoption or consumer purchase behavior of wearable or smart devices because of their value-added features and aesthetic appearance (Yang et al., 2016; Hong et al., 2017; Hsiao and Chen, 2018). Early experimental studies have also found that mental imagery processing can effectively stimulate positive emotions (Kim and Lennon, 2008). A recent study by Ha et al. (2019) investigated and found a significant positive effect of mental imagery on consumers’ positive emotions. Similarly, when the information presented online related to smartwatches is processed by consumers as new features, excellent quality, and a cool appearance through mental imagery, corresponding emotions associated with such imagery are automatically activated, thereby generating positive emotional experiences or responses.

**Hypothesis 3:** Mental imagery will be positively associated with consumers’ positive emotion.

Consumers have difficulties in determining whether to purchase a new product such as a smartwatch in the online shopping context. As such, positive mental imagery encourages consumers to make quick judgments and decisions with limited product information (Slovic et al., 2002, 2007). Mental imagery evoked by concrete pictures in a travel advertisement was found to increase consumers’ behavioral intention to visit this travel website (Miller and Stoica, 2003). Previous research has shown that vivid mental imagery can stimulate a kind of simulated or anticipatory consumption, which helps individuals imagine what the consumption experience would be like and what they would feel (Gavilan et al., 2014). This actual experience resembled by mental imagery not only promotes online consumers’ purchase behavior (Yoo and Kim, 2014) but also motivates consumers’ repurchase intention by providing a virtual experience (Overmars and Poels, 2015). In line with this phenomenon, we expect that the higher the level of mental imagery, the more likely consumers are to make purchasing decisions even without direct product experience on the shopping website. Based on this reasoning, the following hypothesis is proposed:

**Hypothesis 4:** Mental imagery will be positively related to consumers’ purchase intention toward wearable devices.

### Perceived Social Risk and Positive Emotions Affecting Purchase Intention

Social norm theorists argue that expectations from social groups regarding individual behaviors will cause individuals to comply with mainstream social opinions and that individuals will perceive social risk when they anticipate that important peers (e.g., families and friends) will not accept an event or object (Martín et al., 2011). In online shopping settings, perceived social risk refers to the extent to which individuals anticipate that purchased products will not be accepted by important groups such as family members or friends. As a new high-tech product still in the early stage of diffusion (Chuah et al., 2016; Yang et al., 2016), smartwatches are purchased and used only by few innovation adopters (Jeong et al., 2017). Hence, when consumers consider purchasing smartwatches, they are not only concerned about risks related to product function and quality but also interested in important groups’ attitudes toward such new high-tech products. The perceived social norms provide pressures on individuals, which makes them comply with these social norms and form consistent actions with others (Childers and Rao, 1992). Although a few studies have focused on privacy risk (Wu L.-H. et al., 2016), performance and financial risk (Yang et al., 2016), and technological risk (Zhang et al., 2017), little is known regarding the relationship between perceived social risk and consumers’ purchase intention toward smartwatches. Marketing researchers have also suggested that consumers might stop buying a preferred product as a result of disapproval or criticism from families or friends (Hu et al., 1998; Shang et al., 2017). This finding indicates that the consumer purchase decision is influenced by other people’s opinions and that perceived social risk significantly affects consumer decisions (Shang et al., 2017). Neuro-marketing research has further revealed the neural mechanism through which perceived social risk influences individual purchasing decisions and found that the anterior insular implicitly processes an individual’s social risk perception, making consumers refuse to purchase products that are not accepted by social groups (Yokoyama et al., 2014). In line with these studies, it can be reasonably inferred that consumer-perceived social risk of using a smartwatch is negatively associated with his/her intention to purchase it. Thus, the following hypothesis is proposed:

**Hypothesis 5:** Perceived social risk will be negatively associated with consumer intention to purchase a smartwatch.

Positive emotion refers to discrete emotions that we use to describe or express our response to a pleasant experience or object, such as joy, interest, contentment, and love (Fredrickson, 2001). When there is insufficient information to judge the risk of consumer purchasing decision-making, a positive emotional response will increase an individual’s interest and curiosity to explore new products, activate his/her intrinsic motivation to adopt or purchase new products, and further affect their attitude toward them and final behavioral intention (Müllerstewens et al., 2017). According to the affect heuristics, individuals’ emotions can directly influence their final decision (Slovic et al., 2002). Due to the low penetration rate of smartwatches, most consumers have limited knowledge about the attributes of these new products, such as function and quality, and lack feedback on user experience from important groups around them. Recent research has shown that if consumers have to make decisions in the absence of specific cues, emotion will be viewed as an important information source (Pappas et al., 2016; Wu et al., 2017). Generally, the novelty, uniqueness, and aesthetic function and appearance of smartwatches can elicit consumers’ positive emotional responses, such as excitement, surprise, joy, interest, and passion, among others. Prior studies examining online consumer behavior have found that positive emotions can facilitate online purchase behavior, but negative emotions can impede consumers’ purchase intention (Pappas et al., 2014, 2016). Hence, this study proposes that consumers tend to make purchasing decisions if their positive emotions are elicited when browsing pictures, texts, or videos of smartwatches on a shopping website.

**Hypothesis 6:** Positive emotion will be positively associated with consumer intention to purchase a smartwatch.

### Moderation Effect of Individualism–Collectivism

In Hofstede’s culture framework, individualism–collectivism was viewed as value orientations related to a person’s or group’s relationship to others (Hofstede, 1991; Triandis, 1995). Collectivists feel that they belong to a group (Yeniyurt and Townsend, 2003). They tend to pay more attention to group benefits, maintain the integrity of the in-group, and regulate their behavior in light of group norms (Triandis, 1995). Individualists, in contrast, tend to see themselves as independent (Yeniyurt and Townsend, 2003). They have flexible social ties to groups and tend to guide their behavior according to their self-interest (Triandis, 1995), thus giving priority to personal over group goals (Kongsompong et al., 2009). The individualism–collectivism values have been widely employed in cross-national studies, and they appear to be the most extensively investigated dimension in cross-cultural consumer behavioral research (Yeniyurt and Townsend, 2003). For instance, when a new product is launched, it is more easily adopted by consumers in individualistic countries (Yeniyurt and Townsend, 2003). The impact of subjective norms on behavioral intention differed between individualists and collectivists (Srite, 2006; Gupta et al., 2019).

Hofstede’s culture framework was developed to reveal the cultural effect at the country level. However, substantial variations exist in cultural values at the individual level, which may have a critical effect on the individual’s behavior (Srite and Karahanna, 2006; Fang, 2012). The construct’s applicability of Hofstede’s individualism–collectivism at the individual level within nations is relatively scarce (Kongsompong et al., 2009). Particularly, cultural values at the individual level considering significant variations within-nation would be more suitable to studies focusing on individual technology adoption (Fang, 2012). Recent research building on Hofstede’s model has examined the effect of individualism–collectivism at the individual level, advancing our understanding of the effect of cultural values on consumers’ behavior (Srite and Karahanna, 2006; Faqih and Jaradat, 2015). In line with this research stream, this study contributes to both mental imagery and technology adoption research by examining whether individualism–collectivism at the individual level moderates the relationship among mental imagery, perceived social risk, positive emotion, and purchase intention in the context of online shopping for wearable devices.

Hypotheses 2–6 are focused on the mechanism through which mental imagery influences purchase intention by providing evidence for the mediating effects of both perceived social risk and positive emotion. The third purpose of this study was to provide insight into the conditions under which mental imagery plays a greater or reduced role in predicting consumer purchase intention. This study builds on Hofstede’s culture framework by examining how consumers’ individualism–collectivism values moderate the relationship between mental imagery, perceived social risk and positive emotion, and purchase intention.

For socially visible new products such as wearable devices, if the purchase actions conflict with subjective norms, collectivists will experience more social risk than individualists. Consumers holding the collectivism value orientation, therefore, tend to abandon their purchase actions to conform to what the group thinks (Kongsompong et al., 2009). However, consumers holding the individualism value orientation are unlikely to change their decisions, because they are more susceptible to internal attitudes than subjective norms (Bagozzi et al., 2000; Srite, 2006). Thus, we expect perceived social risk to impact the purchase intention toward wearable devices of collectivists with high individualism–collectivism values, but such an effect will not be significant for individualists.

Regarding the influence of positive emotions, it is theorized to represent a type of individuals’ intrinsic motivation and has been found to facilitate purchase intention (Pappas et al., 2014, 2016) or wearable technology adoption (Choi and Kim, 2016; Wu L.-H. et al., 2016; Yang et al., 2016; Cho and Lee, 2017). Following these findings, a positive emotional response or experience can be identified as an individual’s internal benefit. In the context of a wearable device, the positive emotional experience will be regarded as important for individualists because they favor making the purchase decision based on self-interest regardless of others’ ideas and comments (Triandis, 1995). Unlike individualists, such an effect of emotional benefit may not be observed for collectivists because they tend to regard the group over the individual and actively consider opinions from in-group members (Triandis, 1989; Kongsompong et al., 2009). Thus, we expect positive emotion to impact purchase intentions toward wearable devices of individualists, but not collectivists. To summarize, two hypotheses are proposed.

**Hypothesis 7:** Perceived social risk will exhibit a more negative effect on purchase intention for collectivists than individualists.

**Hypothesis 8:** Positive emotion will exhibit a greater effect on purchase intention for collectivists than individualists.

**FIGURE 1:**
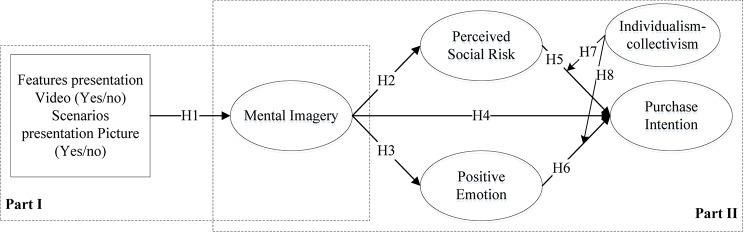
Proposed research model.

Our hypothesized theoretical model is summarized in Figure 1.

## Materials and Methods

### Design and Stimuli

We performed a laboratory experiment to collect data. A two-level (product feature presentation: video vs. text) by two-level (usage scenario presentation: pictures vs. text) between-subject factorial design was developed to validate the research model and verify the hypotheses. Table 1 shows the design conditions.

**TABLE 1:** Treatment conditions.

		**Product feature presentation**	
		**Video**	**Text**
Usage scenario presentation	Picture	Group 1 (*N* = 60)	Group 2 (*N* = 62)
	Text	Group 3 (*N* = 68)	Group 4 (*N* = 64)

We created a simulated online website selling smartwatches for our experiment. Similar to Flavián et al. (2009) and Overmars and Poels (2015), this study considered a real Chinese e-retailer official website as a reference and used it to create this simulated shopping website. For the four treatment conditions, this website was further manipulated with different product presentation conditions: (1) video and picture, (2) text and picture, (3) video and text, and (4) text and text. We manipulated the product feature presentation type by providing video and text. The video downloaded from the reference website contained a real product. Furthermore, product usage scenarios were presented in either concrete pictures or simple text. Each condition had the same semantic information about product features and usage scenarios, ensuring that each experiment website differed only in the product presentation format of product features and usage scenarios.

Following previous research regarding the effect of online product presentation (Park et al., 2005; Algharabat et al., 2017; Flavián et al., 2017), this study chose a smartwatch as the experiment product because it is one of the most popular wearable devices to date, and its price is reasonable for college students. Moreover, a smartwatch is familiar and attractive to the sample population. Furthermore, to control for potential effects of brand on subjects’ psychological and behavioral responses, an actual Chinese smartwatch brand with relatively low popularity—GEAK—was used to increase the ecological validity and legitimacy of the experiment (Overmars and Poels, 2015).

Before the main experiment, a pretest consisting of 78 online shopper samples was conducted to validate whether smartwatches were an appropriate product category to be tested and introduced to the sample population. The results showed that subjects reported that they were familiar with the smartwatch (mean = 4.24, SD = 0.994; six-point Likert scale), but they had little knowledge about the “GEAK” smartwatch (mean = 2.15, SD = 0.939; six-point Likert scale). Further analysis of variance (ANOVA) confirmed that the mean scores of product knowledge about the smartwatch in four cells ranged from 4.13 to 4.38 [*F*_(3,250)_ = 0.78, *p* > 0.1], and the mean scores of brand familiarity to GEAK in four cells ranged from 2.00 to 2.28 [*F*_(3,250)_ = 1.32, *p* > 0.1]. These results suggested that the subjects in all conditions did not vary in product knowledge and brand familiarity, indicating that the experimental stimuli were chosen appropriately.

### Participants

To increase the internal validity due to their homogeneity (Chuah et al., 2016; Jeong et al., 2017), participants were recruited from Anhui University of Technology, a public university with more than 23,000 college students in Anhui Province, located in East China, via advertisements, and they were paid a ¥20 incentive for their participation and collaboration. Specifically, to recruit a relatively large and heterogeneous sample population, researchers posted a recruitment advertisement in the hall of the public library and teaching building with a large flow of people, and provided various instant messaging tools (i.e., WeChat, QQ, and cell phone number) to facilitate interaction with potential subjects. Undergraduate students were chosen as experimental subjects for four reasons: (1) they are one of the major online consumers in China (iResearch, 2015); (2) college students from 18 to 25 years old are the fundamental potential customers of wearable devices (Wu L.-H. et al., 2016; Hsiao and Chen, 2018); (3) many similar studies collected data from college students (Chuah et al., 2016; Jeong et al., 2017); and (4) college student samples may be appropriate to draw conclusions about theory rather than a population (Lee and Gretzel, 2012). All participants in this study had never received any training on the website design, nor did they have any prior practical experience in website design. From recruiting participants on December 6, 2018, to the end of formal experiment, the study lasted for 6 days. The experiment was approved by the Institutional Review Board of the School of Business at the Anhui University of Technology. Each participant was informed of the confidentiality of this study and then provided written informed consent. Finally, a total of 254 students agreed to participate in and completed this study. They were randomly assigned to one of the four conditions; within each condition, cell sizes ranged from 60 to 68. Among 254 valid responses included in the analysis, 49.6% of them were male, with a mean age of 21.27 (SD = 2.25) years, ranging from 18 to 26 years.

### Measures

To develop the questionnaire used in this study, survey items were adapted from previous scales with modifications to fit the specific online shopping context. The original questionnaire was developed in English and then translated into a Chinese version using the back-translation approach. All items were pretested using samples collected from 78 online shoppers. A total of 24 items were retained and included in the final questionnaire. Given that Chinese respondents who are fundamentally influenced by the Zhong-Yong thinking, which emphasizes that one should avoid going to extremes and maintain interpersonal harmony (Chang and Cheng-Ta, 2014; Pan and Sun, 2017), prefer the middle option when filling in the answers, all items were measured with a six-point Likert scale ranging from “1 = strongly disagree” to “6 = strongly agree.” Many researchers also use a 6-point Likert scale (Butler and Wasserman, 2006; Shojania and Mheen, 2015; Schröder et al., 2017), which not only avoids the middle choice but also makes a more detailed distinction in perception and attitude.

Purchase intention (PI) was measured with four items adapted from Hausman and Siekpe (2009) and Grewal et al. (1998). A sample item is “I would consider buying this smartwatch in the near future” (Cronbach’s α = 0.80). Five items with high loadings from the consumption vision scale developed by Walters et al. (2007) were adopted to measure mental imagery (MI) and modified for this study. Sample items are “When I recalled the featured smartwatches, many images that came to my mind were very clear”; “While reviewing the advertisement I found myself daydreaming about the featured smartwatches”; and “This advertisement made me fantasize about having the opportunity to experience the featured smartwatches” (Cronbach’s α = 0.88). Consumers’ perceived social risk (PSR) was measured using four items chosen from Featherman and Pavlou (2003) and Ko et al. (2009). Sample items are “Using a smartwatch might make others have an unfavorable impression of me” and “Using a smartwatch might cause me to lose my reputation” (Cronbach’s α = 0.88). Positive emotion (PE) was measured with six items adapted from the PANAS Scales developed by Watson et al. (1988), such as “excited,” “interested,” “alert,” and “inspired” (Cronbach’s α = 0.89). In this study, individualism–collectivism at the individual level (IC) was measured with five items adapted from Hofstede’s collectivism dimension of cultural values at the individual level developed by Yoo et al. (2011). A sample item is “Individuals should sacrifice self-interest for the group” (Cronbach’s α = 0.96).

### Procedure

At the beginning of the main experiment, participants were randomly assigned to each of four groups. Then, they were asked to read the instructions on the desks ahead of time. In the instructions, all participants were presented with the following specific tasks:

Please imagine you are a white-collar worker with a stable job and income. Recently, a few friends around you discussed smartwatches. Additionally, a few of them have already purchased these really new products. Now, you are planning to buy a smartwatch with a budget of 2,000 RMB. Just then, one of your good friends recommended the GEAK Watch and its online store to you.

Next, the participants were asked to log in to the GEAK official online store and browse the product information for the GEAK smartwatches. After viewing product information about features and usage scenarios in one of four treatment conditions, participants answered all the measures and demographic items.

## Results

### Measurement Model Assessment

Before the reliability test, factor analysis based on principal components extracted common factors and performed orthogonal rotation with the varimax procedure, finally extracting five factors with an eigenvalue greater than 1 and a cumulative explanatory variance explaining 71.9%. All the loadings in Table 2 were greater than 0.60. In the reliability test, the results showed that the normalized Cronbach’s α coefficients of all measures ranged from 0.798 to 0.961 (Table 2), which are greater than the recommended threshold value of 0.70 (Nunnally and Bernstein, 1994), suggesting good internal consistency.

**TABLE 2:** Results of EFA and CFA.

**Variables**	**Items**	**Factors**					**AVE**	**CR**
		**1**	**2**	**3**	**4**	**5**		
Positive emotion	This smartwatch makes me interested.	0.156	0.729	0.269	−0.154	0.105	0.576	0.891
	This smartwatch makes me excited.	0.043	0.765	0.204	−0.175	0.208		
	This smartwatch makes me inspired.	0.075	0.743	0.135	−0.024	0.151		
	This smartwatch makes me alert.	0.138	0.778	0.144	−0.053	0.064		
	This smartwatch makes me involved.	0.168	0.768	0.096	−0.089	0.221		
	This smartwatch makes me fascinated.	0.177	0.730	0.257	−0.138	0.038		
Individualism-collectivism	Individuals should sacrifice self-interest for the group.	0.871	0.163	0.108	−0.142	0.214	0.838	0.963
	Group welfare is more important than individual rewards.	0.891	0.134	0.088	−0.152	0.182		
	Group success is more important than individual success.	0.872	0.169	0.061	−0.181	0.196		
	Individuals should only pursue their goals after considering the welfare of the group.	0.887	0.112	0.123	−0.129	0.212		
	Group loyalty should be encouraged even if individual goals suffer.	0.863	0.148	0.084	−0.152	0.209		
Mental imagery	When I recalled the featured smartwatches, many images that came to my mind were very clear.	0.097	0.317	0.753	−0.154	0.052	0.597	0.881
	When I recalled the featured smartwatches, many images that came to my mind were very vivid.	0.172	0.165	0.808	−0.163	0.155		
	The mental images that came to mind made me feel as though I was actually experiencing this smartwatch featured in this advertisement.	0.153	0.116	0.705	−0.297	0.208		
	While reviewing the advertisement, I found myself daydreaming about the featured smartwatches.	0.004	0.258	0.762	−0.119	0.088		
	This advertisement made me fantasize about having an opportunity to experience the featured smartwatches.	0.052	0.246	0.685	−0.231	0.317		
Perceived social risk	Using a smartwatch might make others have an unfavorable impression of me.	−0.146	−0.090	−0.144	0.833	−0.001	0.643	0.878
	Using a smartwatch might cause me to lose my reputation.	−0.188	−0.094	−0.201	0.810	−0.170		
	Using a smartwatch might negatively affect the way others think of me.	−0.142	−0.106	−0.186	0.812	−0.155		
	Using a smartwatch might lead to a social loss for me because my relatives and friends would think less highly of me.	−0.167	−0.207	−0.236	0.745	−0.073		
Purchase intention	I would consider buying this smartwatch in the near future.	0.154	0.277	0.244	−0.103	0.673	0.534	0.82
	The probability that I would buy this smartwatch is high.	0.339	0.230	0.108	−0.076	0.676		
	I expect to purchase this smartwatch in the near future.	0.317	0.124	0.155	−0.068	0.746		
	It is likely that I would purchase this smartwatch in the near future.	0.336	0.122	0.216	−0.225	0.602		

*AVE, average variance extraction; CR, composite reliability.*

The confirmatory factor analysis was used to test two types of commonly reported construct validity, namely, convergent and discriminant validity. An examination of the measurement model fit statistics suggested an acceptable goodness-of-fit (*χ2/df* = = 1.521, RMR = 0.044, AGFI = 0.873, IFI = 0.970, CFI = 0.970, RMSEA = 0.045). The convergent validity results presented in Table 2 show that the average variance extraction (AVE) values of all latent variables (0.534∼0.838) were greater than the expected cutoff value of 0.50 (Fornell and Larcker, 1981). The composite reliability (CR) ranged from 0.819 to 0.963, and it exceeded the threshold of 0.7 (Nunnally and Bernstein, 1994). Hence, the AVEs and CRs of all constructs satisfied the threshold values, providing evidence of high convergent validity.

According to Fornell and Larcker (1981), the square root of AVE values should be higher than their inter-construct correlations to achieve discriminant validity. The results shown in Table 3 indicate that the discriminant validity of all constructs was verified.

**TABLE 3:** Square roots of AVEs and correlations.

	**MI**	**PSR**	**PI**	**PE**	**IC**
MI	0.773				
PSR	−0.505	0.802			
PI	0.507	−0.392	0.731		
PE	0.540	−0.355	0.482	0.759	
IC	0.323	−0.400	0.612	0.374	0.915

*Values on the diagonal line are the square roots of the average variance extracted for each construct. MI, mental imagery; PSR, perceived social risk; PI, purchase intention; PE, positive emotion; IC, individualism–collectivism at the individual level.*

### Hypothesis Testing

**FIGURE 2:**
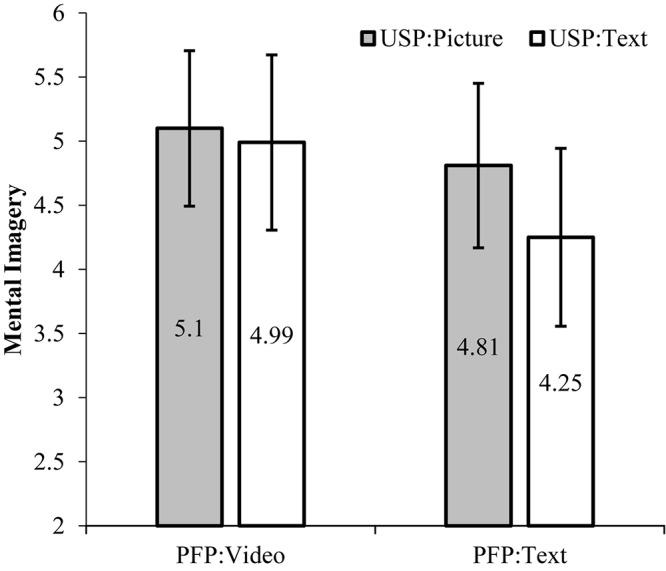
Difference in mental imagery by the feature presentation video and scenario presentation picture. PFP, product feature presentation; USP, usage scenario presentation.

The ANOVAs were performed using the product feature condition (video vs. text) and usage scenario (picture vs. text) to test Hypothesis 1 (H1), which investigates the effects of online product presentation on mental imagery (part I in Figure 1). A significant main effect of the product feature condition on mental imagery was confirmed [*M*_video_ = 5.05, *SD* = 0.65 vs. *M*_no–video_ = 4.53, *SD* = 0.72; *F*_(3, 250)_ = 39.336, *p* < 0.001, partial *η^2^* = 0.136]. Additionally, the same main effect was found in the usage scenario condition [*M*_picture_ = 4.96, *SD* = 0.64 vs. *M*_no–picture_ = 4.63, *SD* = 0.78; *F*_(1,252)_ = 16.657, *p* < 0.001, partial *η^2^* = 0.062]. The interaction was found to be significant [*F*_(3,250)_ = 7.623, *p* < 0.01, partial *η^2^* = 0.030] and was driven by differences in the related condition (see Figure 2). The difference in mean scores of mental imagery between scenario presentation conditions was not significant when the product features were exposed in the video [*M*_picture_ = 5.10, *SD* = 0.61 vs. *M*_no–picture_ = 4.99, *SD* = 0.68; *F*_(1,126)_ = 0.94, *p* > 0.1]. However, when the product features were presented as text (no video), the difference in mental imagery was distinct between the two scenario presentation conditions [*M*_picture_ = 4.81, *SD* = 0.64 vs. *M*_no–picture_ = 4.25, *SD* = 0.69; *F*_(1,124)_ = 22.58, *p* < 0.001]. Therefore, the results strongly support H1.

**FIGURE 3:**
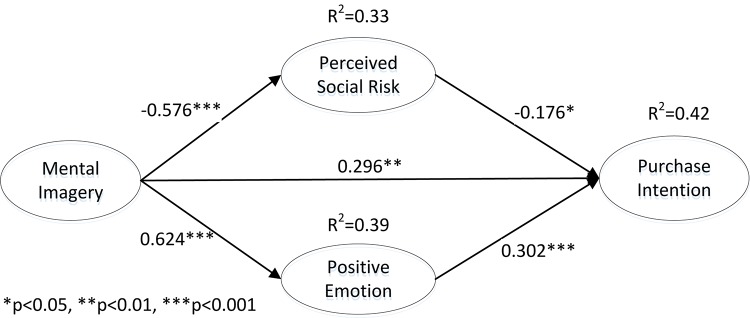
Results for the structural model (full sample).

The structural equation modeling approach with AMOS 17.0 was used to test H2 through H6, which examine both direct and indirect effects of mental imagery on purchase intention (part II in Figure 1). According to the threshold value (Hu et al., 1998), all examined goodness-of-fit indices of the structural model were satisfactory: χ2/*df* = 1.488, RMR = 0.046, AGFI = 0.895, IFI = 0.972, CFI = 0.972, and RMSEA = 0.044, suggesting that the proposed structural model fit well with the data. The results also provided support for the hypothesized relationships, and the model could explain more than 42% of the variance in purchase intention. Figure 3 shows the standardized parameter estimation results for the hypothesized model.

As hypothesized, mental imagery was found to not only reduce consumers’ perceived social risk (β = −0.576, *p* < 0.001) but also trigger positive emotion (β = 0.624, *p* < 0.001), strongly supporting H2 and H3, respectively. In addition, mental imagery was associated with purchase intention (β = 0.296, *p* < 0.01), indicating that H4 was supported. As expected, the results showed that the perceived social risk (β = −0.176, *p* < 0.05) and positive emotion (β = 0.302, *p* < 0.001) exhibited significant effects on consumers’ purchase intention, thus providing evidence for H5 and H6.

### Moderating Effect Testing

We conducted a multigroup analysis to test the moderating role of individualism–collectivism at the individual level (H7 and H8). The full sample was divided into two data sets by K-medoids clustering (median = 3.20): individualist group (*N* = 125) and collectivist group (N = 129) (*M*_Collectivist_ = 4.02, *SD*_Collectivist_ = 0.76 vs. *M*_Individualist_ = 1.89, *SD*_Individualist_ = 0.50; *t* (254) = 26.18, *p* < 0.001). Then, we used AMOS 17.0 to estimate the same research model with the two subgroup data sets. As shown in Table 4, for the structural weights model (M3), there was a significant difference in structural weights between collectivists and individualists, with Δ*χ*^2^(Δ*df*) of 30.216(5) and a *p*-value of 0.000.

**TABLE 4:** Results of the multigroup analysis.

**Model**	**χ^2^**	**df**	**χ^2^/df**	**RMR**	**CFI**	**RMSEA**	**Δχ^2^ (Δdf)**	***p***
M_I_	181.281	144	1.259	0.050	0.966	0.045	–	–
M_G_	175.651	144	1.220	0.056	0.970	0.042	–	–
M_1_	356.933	288	1.239	0.053	0.968	0.031	–	–
M_2_	374.694	303	1.237	0.061	0.967	0.031	17.762 (15)	0.275
M_3_	404.911	308	1.315	0.076	0.955	0.035	30.216 (5)	0.000

*M_*I*_ is the model of the collectivist group; M_*G*_ is the model of the individualist group; M_1_ is the unconstrained model; M_2_ is the measurement weights model; M_3_ is the structural weights model.*

Further, we examined the critical ratios for differences between parameters (CRDPs) to assess the between-group differences in each path coefficient, with a z-score of greater than 1.96 considered significantly different for the subgroups (Simons and Kaczynski, 2012). The CRDPs, reported in Table 5, suggested that the PSR–PI link was significantly different between the collectivist and individualist subgroups, with a CRDP of −2.230. The results of the multigroup analysis indicated that perceived social risk had a significant negative influence on collectivists’ purchase intention (β_Collectivist_ = −0.252, *p* < 0.05). However, this effect was not significant for individualists (β_Individualist_ = 0.077, *p* > 0.05). Although the influence of positive emotion on purchase intention was different between collectivists (β_Collectivist_ = 0.172, *p* > 0.05) and individualists (β_Individualist_ = 0.399, *p* < 0.05), this difference was not significant, with a CRDP of −0.605. In summary, the results support H7 but not H8.

**TABLE 5:** Critical ratios for differences between parameters (unconstrained).

	**b1_1**	**b2_1**	**b3_1**	**b4_1**	**b5_1**
b1_2	−1.267	2.331	3.99	7.489	3.162
b2_2	−4.067	−0.605	1.014	5.796	0.562
b3_2	−6.37	−3.416	−2.23	4.081	−1.918
b4_2	−6.869	−4.554	−3.764	1.501	−3.481
b5_2	−2.421	1.176	2.864	6.814	2.13

*b1 = MI–PE, b2 = PE–PI, b3 = PSR–PI, b4 = MI–PSR, b5 = MI–PI. “_1” and “_2” represent the individualist and collectivist group, respectively. If the critical ratio for differences between parameters (CRDP) value on the diagonal is greater than 1.96, it indicates that there is a significant difference between the two groups.*

### Additional Analysis

The moderating effect of gender on information technology adoption has attracted considerable attention. The results, however, are inconsistent. For example, an influential study by Venkatesh et al. (2012) found that, when considering use of a new technology, women tend to place greater emphasis on external supporting factors, whereas men tend to rely less on facilitating conditions. Instead, other researchers reported that male and female users did not demonstrate significantly different behaviors in adopting or using mobile technology for shopping (Bigne et al., 2005; Faqih and Jaradat, 2015). To further test whether our findings reported in Figure 3 vary between men and women, we used AMOS 17.0 to estimate the same research model with the two subgroup data sets (*N*_men_ = 126 vs. *N*_women_ = 128). The results, reported in Table 6, suggested that except for the influence of mental imagery on purchase intention, gender was not found to moderate the hypothesized relationships. The findings support the conclusions of Bigne et al. (2005) and Faqih and Jaradat (2015).

**TABLE 6:** Structural model result comparisons between men and women.

			**Men (*N* = 126)**			**Women (*N* = 128)**		
			**Estimate**	**CR**	***p***	**Estimate**	**CR**	***p***
PE	<—	MI	0.645	6.103	<0.001	0.635	5.469	<0.001
PSR	<—	MI	−0.847	−5.772	<0.001	−0.538	−4.782	<0.001
PI	<—	PE	0.229	2.628	0.009	0.185	2.583	0.010
PI	<—	PSR	−0.107	−1.837	0.066	−0.109	−1.632	0.103
PI	<—	MI	0.276	2.591	0.010	0.156	1.821	0.069

## Discussion

The purpose of this study was to examine how and when online product presentation influences purchase intention toward wearable devices. The results show that usage scenario presentation as a marketing element affects mental imagery, such that consumers experience greater mental imagery when they are exposed to concrete pictures rather than to textual content. More importantly, this effect holds true only when the features of wearable devices are presented in text-without-video format. Furthermore, we show that in addition to a direct effect, mental imagery plays an indirect role in increasing purchase intention by reducing the perceived social risk and increasing positive emotion, but these effects of mental imagery are moderated by individualism–collectivism at the individual level. Further, the findings show that males and females do not demonstrate variation in intention to purchase wearable devices, which is in line with some prior results.

This research has contributed to online product presentation, mental imagery, and technology adoption. First, we advance the understanding of the effect of two different stimuli on mental imagery in the context of buying wearable devices online. Most of the previous mental imagery processing research has focused on print advertising and usually included one external advertising stimulus (Babin and Burns, 1997; Lee and Gretzel, 2012). Theoretical research examining the interaction effects of more different stimuli, specifically in the context of a shopping website, has remained unexplored. This study contributes to both online product presentation and mental imagery research by simultaneously investigating the interaction effects of two different stimuli, i.e., product feature presentation video and usage scenario presentation picture, in the context of purchasing wearable devices (a type of new high-tech product). Prior research has confirmed the role of the dynamic video and/or concrete pictures in evoking individual mental imagery (Babin and Burns, 1997; Lee and Gretzel, 2012; Wu K. et al., 2016; Orús et al., 2017). The present findings demonstrated that in addition to these accounts, an interaction effect between two stimuli also occurs, such that the imagery effect of usage scenario presentation pictures is significant only for consumers exposed to textual product feature information. A possible reason for this finding is that a large quantity of mental imagery has been stimulated by vivid and rich product information provided by the dynamic presentation video, which makes concrete pictures ineffective (Babin and Burns, 1997).

Second, this study contributes to the body of knowledge by examining how mental imagery drives consumers’ intention to purchase wearable devices. From the perspective of imagery processing, researchers have highlighted the importance of mental imagery processing in the context of online shopping (Schlosser, 2003; Gavilan et al., 2014; Flavián et al., 2017), but relatively little is known about the mediators linking mental imagery and purchase intention. Although few scholars have explored the mediating effects of experience value (Overmars and Poels, 2015) and positive emotion (Yoo and Kim, 2014), it is unclear if mental imagery motivates purchase intention by reducing consumers’ perception of shopping risk. The results from this study extend the mental imagery literature by shedding light on the mediating effect of the perceived social risk that usually occurs when buying a new product such as a wearable device, supported by prior findings. In contrast, this study enriches the theoretical basis for wearable technology adoption by showing the effectiveness of evoking consumers’ imagery processing and perceived social risk. Mental imagery has been widely examined in consumer behavioral literature, but little attention has previously been paid by researchers of information systems (Wu and Holsapple, 2014). Regarding wearable technology adoption, while the effects of individual cognitive and emotional responses, e.g., perceived ease of use, perceived usefulness, social influence, and emotion, on behavioral intention have been found in recent studies (Choi and Kim, 2016; Wu L.-H. et al., 2016; Yang et al., 2016; Cho and Lee, 2017), the potential impact of perceived social risk remains unknown. This study shows that in addition to an indirect effect, mental imagery can increase individual behavioral intention toward wearable technologies by decreasing the perceived social risk and eliciting positive emotional responses.

Third, this study extends the existing research on both mental imagery and technology adoption by demonstrating the moderating effect of individualism–collectivism at the individual level in the context of online buying of wearable devices. With the increased focus on the impact of imagery processing in consumer behavioral intention (Schlosser, 2003; Yoo et al., 2011; Overmars and Poels, 2015; Orús et al., 2017), very little is known about the conditions under which mental imagery strengthens or weakens purchase intention. The most interesting finding of this study is that mental imagery has a greater effect on purchase intention for collectivists than individualists. Specifically, extending previous findings that mental imagery is correlated with behavioral intention (Overmars and Poels, 2015; Orús et al., 2017) to a new domain, our research reveals that mental imagery is likely to be more effective in generating purchase intention for collectivists. In addition, this study contributes significantly to the expansion of technology adoption research by showing the cultural moderating effect on consumers’ behavioral intention toward wearable devices. Although the effect of perceived risk on individual intention to adopt new technologies or products has been confirmed in information systems and consumer behavioral literature (Featherman and Pavlou, 2003; Park et al., 2005; Yang et al., 2016; Shang et al., 2017; Zhang et al., 2017), little attention has been paid to examining the moderating effect of individualism–collectivism at the individual level on such phenomena (Kongsompong et al., 2009; Faqih and Jaradat, 2015). Following recent research focusing on the effects of individualism–collectivism at the within-nation individual level (Srite and Karahanna, 2006; Kongsompong et al., 2009; Fang, 2012; Faqih and Jaradat, 2015; Gupta et al., 2019), this study tested individualism–collectivism at the individual level as a moderator and demonstrated that although perceived social risk mediates the relationship between mental imagery and purchase intention, such a mediating effect is significant only for the collectivist, and not the individualist.

## Limitations and Future Directions

Whereas the findings of this study are valid and valuable, the results must be approached with caution for several reasons. First and most importantly, the study design was limited by focusing only on two cognitive and emotional variables. Future studies should include other cognitive and emotional variables, such as perceived usefulness, perceived relative advantage, service excellence, and perceived quality, in the framework of the present study, which will provide a better understanding of how consumers respond to online smartwatch presentation. Second, this study showed the interaction effect of two different online presentation stimuli, but it was limited by underplaying the boundary conditions, which might dilute the relationship. For example, previous studies have suggested that the style of processing moderates the relationship between the concreteness of pictures and mental imagery (Yoo and Kim, 2014), and information load moderates the relationship between online product presentation and online shopping performance (Li et al., 2016). Thus, future research should explicitly identify and test possible moderators of the relationship between online product presentation and purchase intention. Third, the samples were collected only in Chinese populations, particularly college students, and thus, the generalizability of the findings could be limited. Generalization of the model’s scope would require a global data collection process for thorough validation. Future studies could investigate a larger and more diverse cross-section of the population, using stratified or quota sampling to ensure a reasonable distribution of demographic variables. Altogether, these limitations imply the need for more rigorous methods and theoretical refinement. Future research will benefit from replicating or extending the current study with a more diverse group of subjects for generalizability. Lastly, similar to Patterson et al. (2006), Yoo et al. (2011), Faqih and Jaradat (2015), and Hallikainen and Laukkanen (2018), the present study only used the Collectivistic Scale to evaluate individualism–collectivism at the individual level. We believe that future research can benefit from re-examining our research model with both scales.

## Data Availability Statement

The datasets generated for this study are available on request to the corresponding author.

## Ethics Statement

The studies involving human participants were reviewed and approved by the Institutional Review Board of the School of Business at Anhui University of Technology. The patients/participants provided their written informed consent to participate in this study.

## Author Contributions

JW contributed to the conceptualization, formal analysis, funding acquisition, and investigation. FW contributed to the methodology and wrote the first draft of the manuscript. LL contributed to the formal analysis, investigation, and writing–editing. DS made critical revisions of the manuscript.

## Conflict of Interest

The authors declare that the research was conducted in the absence of any commercial or financial relationships that could be construed as a potential conflict of interest.

## References

[B1] Algharabat R. Abdallah Alalwan A. Rana N. P. Dwivedi Y. K. (2017). Three dimensional product presentation quality antecedents and their consequences for online retailers: the moderating role of virtual product experience. *J. Retail. Consum. Serv.* 36 203–217. 10.1016/j.jretconser.2017.02.007

[B2] Babin B. J. Griffin M. Borges A. Boles J. S. (2013). Negative emotions, value and relationships: differences between women and men. *J. Retail. Consum. Serv.* 20 471–478. 10.1016/j.jretconser.2013.04.007 9637336

[B3] Babin L. A. Burns A. C. (1997). Effects of print ad pictures and copy containing instructions to imagine on mental imagery that mediates attitudes. *J. Advert.* 26 33–44. 10.1080/00913367.1997.10673527

[B4] Bagozzi R. P. Wong N. Abe S. Bergami M. (2000). Cultural and situational contingencies and the theory of reasoned action: application to fast food restaurant consumption. *J. Cons. Psychol.* 9 97–106. 10.1207/15327660051044187

[B5] Bigne E. Ruiz C. Sanz S. (2005). The impact of internet user shopping patterns and demographics on consumer mobile buying behaviour. *J. Electr. Commer. Res.* 6 193–210.

[B6] Burns A. C. Biswas A. Babin L. A. (1993). The operation of visual imagery as amediator of advertising effects. *J. Advert.* 22 71–85. 10.1080/00913367.1993.10673405

[B7] Butler B. Wasserman A. W. (2006). The role of death qualification in Venirepersons’ attitudes toward the insanity defense. *J. Appl. Soc. Psychol.* 36 1744–1757. 10.1111/j.0021-9029.2006.00079.x

[B8] Chang T. Y. Cheng-Ta Y. (2014). Individual differences in Zhong-Yong tendency and processing capacity. *Front. Psychol.* 5:1316. 10.3389/fpsyg.2014.01316 25477842 PMC4237047

[B9] Childers T. L. Rao A. R. (1992). The influence of familial and peer-based reference groups on consumer decisions. *J. Consum. Res.* 19 198–211.

[B10] Cho J. Lee H. E. (2017). Contextualization of motivations determining the continuance intention to use smart devices among people with physical disabilities. *Telemat. Inform.* 34 338–350. 10.1016/j.tele.2016.05.011

[B11] Choi J. Kim S. (2016). Is the smartwatch an IT product or a fashion product? A study on factors affecting the intention to use smartwatches. *Comput. Hum. Behav.* 63 777–786. 10.2196/10238 30672745 PMC6366393

[B12] Chuah H. W. Rauschnabel P. A. Krey N. Bang N. Ramayah T. Lade S. (2016). Wearable technologies: the role of usefulness and visibility in smartwatch adoption. *Comput. Hum. Behav.* 65 276–284. 10.1016/j.chb.2016.07.047

[B13] Davis F. D. Bagozzi R. P. Warshaw P. R. (1989). User acceptance of computer technology: a comparison of two theoretical models. *Manag. Sci.* 35 982–1003. 10.1287/mnsc.35.8.982

[B14] Fang T. (2012). Yin yang: a new perspective on culture. *Manag. Org. Rev.* 8 25–50. 10.1111/j.1740-8784.2011.00221.x

[B15] Faqih K. M. S. Jaradat M. I. R. M. (2015). Assessing the moderating effect of gender differences and individualism-collectivism at individual-level on the adoption of mobile commerce technology: TAM3 perspective. *J. Retail. Consum. Serv.* 22 37–52. 10.1016/j.jretconser.2014.09.006

[B16] Featherman M. S. Pavlou P. A. (2003). Predicting e-services adoption: a perceived risk facets perspective. *Int. J. Hum. Comput. Stud.* 59 451–474. 10.1016/s1071-5819(03)00111-3

[B17] Flavián C. Gurrea R. Orús C. (2009). The effect of product presentation mode on the perceived content and continent quality of web sites. *Online Inform. Rev.* 33 1103–1128. 10.1108/14684520911011034

[B18] Flavián C. Gurrea R. Orús C. (2017). The influence of online product presentation videos on persuasion and purchase channel preference: the role of imagery fluency and need for touch. *Telemat. Inform.* 34 1544–1556. 10.1016/j.tele.2017.07.002

[B19] Fornell C. Larcker D. (1981). Evaluating structure equations models with unobservable variables and measurement error. *J. Mark. Res.* 18 39–50. 10.1177/002224378101800104

[B20] Fredrickson B. L. (2001). The role of positive emotions in positive psychology: the broaden-and-build theory of positive emotions. *Am. Psychol.* 56 218–226. 10.1037/0003-066x.56.3.218 11315248 PMC3122271

[B21] Gavilan D. Avello M. Abril C. (2014). The mediating role of mental imagery in mobile advertising. *Int. J. Inform. Manag.* 34 457–464. 10.1016/j.ijinfomgt.2014.04.004

[B22] Goossens C. (2000). Tourism information and pleasure motivation. *Ann. Tourism Res.* 27 301–321. 10.1016/s0160-7383(99)00067-5

[B23] Grewal D. Krishnan R. Baker J. Borin N. (1998). The effect of store name, brand name and price discounts on consumers’ evaluations and purchase intentions. *J. Retail.* 74 331–352. 10.1016/s0022-4359(99)80099-2

[B24] Gu Z. Wei J. Xu F. (2016). An empirical study on factors influencing Consumers’ initial trust in wearable commerce. *J. Comput. Inform. Syst.* 56 79–85. 10.1080/08874417.2015.11645804

[B25] Gupta M. Esmaeilzadeh P. Uz I. Tennant V. M. (2019). The effects of national cultural values on individuals’ intention to participate in peer-to-peer sharing economy. *J. Bus. Res.* 97 20–29. 10.1016/j.jbusres.2018.12.018

[B26] Ha S. Huang R. Park J.-S. (2019). Persuasive brand messages in social media: a mental imagery processing perspective. *J. Retail. Consum. Serv.* 48 41–49. 10.1016/j.jretconser.2019.01.006

[B27] Hallikainen H. Laukkanen T. (2018). National culture and consumer trust in e-commerce. *Int. J. Inform. Manag.* 38 97–106. 10.1016/j.ijinfomgt.2017.07.002

[B28] Hausman A. V. Siekpe J. S. (2009). The effect of web interface features on consumer online purchase intentions. *J. Bus. Res.* 62 5–13. 10.1016/j.jbusres.2008.01.018

[B29] Hofstede G. (1991). *Cultures and Organizations: Software of the Mind.* New York, NY: McGraw Hill.

[B30] Hong J. C. Lin P. H. Hsieh P. C. (2017). The effect of consumer innovativeness on perceived value and continuance intention to use smartwatch. *Comput. Hum. Behav.* 67 264–272. 10.1016/j.chb.2016.11.001

[B31] Hsiao K. L. Chen C. C. (2018). What drives smartwatch purchase intention? Perspectives from hardware, software, design, and value. *Telemat. Inform.* 35 103–113. 10.1016/j.tele.2017.10.002

[B32] Hu L. Bentler P. Hu L. (1998). Fit indices in covariance structure modelling: sensitivityto underparameterization model misspecification. *Psychol. Methods* 3 424–453. 10.1037/1082-989x.3.4.424

[B33] iResearch (2015). *RE: Market Status and Core Players’ Strategies of China Intelligent Hardware Industry Research Series.* Pune: iResearch.

[B34] Jeong S. C. Kim S. H. Ji Y. P. Choi B. (2017). Domain-specific innovativeness and new product adoption: a case of wearable devices. *Telemat. Inform.* 34 399–412. 10.1016/j.tele.2016.09.001

[B35] Karahanna E. Straub D. W. Chervany N. L. (1999). Information technology adoption across time: a cross-sectional comparison of pre-adoption and post-adoption beliefs. *MIS Q.* 23 183–213.

[B36] Ki C. Lee K. Kim Y.-K. (2017). Pleasure and guilt: how do they interplay in luxury consumption? *Eur. J. Mark.* 51 722–747. 10.1108/ejm-07-2015-0419

[B37] Kim M. Lennon S. (2008). The effects of visual and verbal information on attitudes and purchase intentions in internet shopping. *Psychol. Mark.* 25 146–178. 10.1002/mar.20204

[B38] Ko E. Sung H. Yun H. (2009). Comparative analysis of purchase intentions toward smart clothing between Korean and U.S. *Consum. Cloth. Text. Res. J.* 27 259–273. 10.1177/0887302x08327086

[B39] Kongsompong K. Green R. T. Patterson P. G. (2009). Collectivism and social influence in the buying decision: a four-country study of inter- and intra-national differences. *Aust. Mark. J.* 17 142–149. 10.1016/j.ausmj.2009.05.013

[B40] Lee S. Y. Lee K. (2018). Factors that influence an individual’s intention to adopt a wearable healthcare device: The case of a wearable fitness tracker. *Technol. Forecast. Soc. Change* 129 154–163. 10.1016/j.techfore.2018.01.002

[B41] Lee W. Gretzel U. (2012). Designing persuasive destination websites: a mental imagery processing perspective. *Tour. Manag.* 33 1270–1280. 10.1016/j.tourman.2011.10.012

[B42] Li M. Wei K.-K. Tayi G. K. Tan C.-H. (2016). The moderating role of information load on online product presentation. *Inform. Manag.* 53 467–480. 10.1016/j.im.2015.11.002

[B43] MacInnis D. J. Price L. L. (1987). The role of imagery in information processing: review and extensions. *J. Consum. Res.* 13 473–491.

[B44] Martín S. S. Camarero C. José R. S. (2011). Dual effect of perceived risk on cross-national e-commerce. *Internet Res.* 21 46–66. 10.1108/10662241111104875

[B45] Miller D. W. Stoica M. (2003). Comparing the effects of a photograph versus artistic renditions of a beach scene in a direct response print ad for a Caribbean resort island: a mental imagery perspective. *J. Vacat. Mark.* 10 11–21. 10.1177/135676670301000102

[B46] Müllerstewens J. Schlager T. Häubl G. Herrmann A. (2017). Gamified information presentation and consumer adoption of product innovations. *J. Mark.* 81 8–24. 10.1509/jm.15.0396

[B47] Nascimento B. Oliveira T. Tam C. (2018). Wearable technology: what explains continuance intention in smartwatches? *J. Retail. Consum. Serv.* 43 157–169. 10.1016/j.jretconser.2018.03.017

[B48] Nunnally J. Bernstein I. (1994). *Psychometric Theory (3rd).* New York: McGraw-Hill.

[B49] Orús C. Gurrea R. Flavián C. (2017). Facilitating imaginations through online product presentation videos: effects on imagery fluency, product attitude and purchase intention. *Electr. Commer. Rese.* 17 661–700. 10.1007/s10660-016-9250-7

[B50] Overmars S. Poels K. (2015). How product representation shapes virtual experiences and re-patronage intentions: the role of mental imagery processing and experiential value. *Int. Rev. Retail Distribut. Consum. Res.* 25 236–259. 10.1080/09593969.2014.988279

[B51] Pan W. Sun L.-Y. (2017). A self-regulation model of Zhong Yong thinking and employee adaptive performance. *Manag. Organ. Rev.* 14 1–25.

[B52] Pappas I. O. Kourouthanassis P. E. Giannakos M. N. Chrissikopoulos V. (2014). Shiny happy people buying: the role of emotions on personalized e-shopping. *Electr. Markets* 24 193–206. 10.1007/s12525-014-0153-y

[B53] Pappas I. O. Kourouthanassis P. E. Giannakos M. N. Chrissikopoulos V. (2016). Explaining online shopping behavior with fsQCA: the role of cognitive and affective perceptions. *J. Bus. Res.* 69 794–803. 10.1016/j.jbusres.2015.07.010

[B54] Park J. Lennon S. J. Stoel L. (2005). On-line product presentation: effects on mood, perceived risk, and purchase intention. *Psychol. Mark.* 22 695–719. 10.1002/mar.20080

[B55] Patterson P. G. Cowley E. Prasongsukarn K. (2006). Service failure recovery: the moderating impact of individual-level cultural value orientation on perceptions of justice. *Int. J. Res. Mark.* 23 263–277. 10.1016/j.ijresmar.2006.02.004

[B56] Pavlou P. A. (2003). Consumer acceptance of electronic commerce : integrating trust and risk with the technology acceptance model. *Int. J. Electr. Commer.* 7 69–103.

[B57] Petrova P. K. Cialdini R. B. (2005). Fluency of consumption imagery and the Backfire effects of imagery appeals. *J. Consum. Res.* 32 442–452. 10.1086/497556

[B58] Rodríguez-Ardura I. Meseguer-Artola A. (2015). e-Learning continuance: the impact of interactivity and the mediating role of imagery, presence and flow. *Inform. Manag.* 53 504–516. 10.1016/j.im.2015.11.005

[B59] San Martín S. Camarero C. San José R. (2011). Dual effect of perceived risk on cross-national e-commerce. *Internet Res.* 21 46–66. 10.1108/10662241111104875

[B60] Schlosser A. E. (2003). Experiencing products in the virtual world: the role of goal and imagery in influencing attitudes versus purchase intentions. *J. Consum. Res.* 30 184–198. 10.1086/376807

[B61] Schröder H. Henke A. Stieger L. Beckers S. Sopka S. (2017). Influence of learning styles on the practical performance after the four-step basic life support training approach – An observational cohort study. *PLoS One* 12:e0178210. 10.1371/journal.pone.0178210 28542636 PMC5439953

[B62] Shang Q. Pei G. Jin J. (2017). My friends have a word for it: event-related potentials evidence of how social risk inhibits purchase intention. *Neurosci. Lett.* 643 70–75. 10.1016/j.neulet.2017.02.023 28215877

[B63] Shin D. Biocca F. (2018). Impact of social influence and users’ perception of coolness on smartwatch behavior. *Soc. Behav. Pers.* 46 881–890. 10.2224/sbp.5134

[B64] Shojania K. G. Mheen M. V. D. (2015). Temporal trends in patient safety in the Netherlands: reductions in preventable adverse events or the end of adverse events as a useful metric? *BMJ Qual. Saf.* 24 541–544. 10.1136/bmjqs-2015-004461 26150549

[B65] Simons L. E. Kaczynski K. J. (2012). The fear avoidance model of chronic pain: examination for pediatric application. *J. Pain* 13 827–835. 10.1016/j.jpain.2012.05.002 22832693 PMC3438388

[B66] Slovic P. Finucane M. Peters E. GMacGregor D. (2002). Rational actors or rational fools: implications of the affect heuristic for behavioral economics. *J. Soc. Econ.* 31 329–342. 10.1016/s1053-5357(02)00174-9

[B67] Slovic P. Finucane M. L. Peters E. Macgregor D. G. (2007). The affect heuristic. *Eur. J. Operat. Res.* 177 1333–1352.

[B68] Sobkow A. Traczyk J. Zaleskiewicz T. (2016). The affective bases of risk perception: negative feelings and stress mediate the relationship between mental imagery and risk perception. *Front. Psychol.* 7:932. 10.3389/fpsyg.2016.00932 27445901 PMC4919331

[B69] Srite M. (2006). Culture as an explanation of technology acceptance differences: an empirical investigation of Chinese and US users. *Aus. J. Inform. Syst.* 14 30–52.

[B70] Srite M. Karahanna E. (2006). The role of espoused national cultural values in technology acceptance. *MIS Q.* 30 679–704.

[B71] Triandis H. C. (1989). The self and social behavior in differing cultural contexts. *Psychol. Rev.* 96 506–520. 10.1037/0033-295x.96.3.506

[B72] Triandis H. C. (1995). *Individualism and Collectivism.* Boulder: Westview Press.

[B73] Venkatesh V. (2000). Determinants of perceived ease of use: integrating control, intrinsic motivation, and emotion into the technology acceptance model. *Inform. Syst. Res.* 11 342–365. 10.1287/isre.11.4.342.11872

[B74] Venkatesh V. Thong J. Y. L. Xu X. (2012). Consumer acceptance and use of information technology: extending the unified theory of acceptance and use of technology. *MIS Q.* 36 157–178.

[B75] Walters G. Sparks B. Herington C. (2007). The effectiveness of print advertising stimuli in evoking elaborate consumption visions for potential travelers. *J. Travel Res.* 46 24–34. 10.1177/0047287507302376

[B76] Watson D. Clark L. A. Tellegen A. (1988). Development and validation of brief measures of positive and negative affect: the PANAS scales. *J. Pers. Soc. Psychol.* 54 1063–1070. 10.1037/0022-3514.54.6.1063 3397865

[B77] Wu J. Holsapple C. (2014). Imaginal and emotional experiences in pleasure-oriented IT usage: a hedonic consumption perspective. *Inform. Manag.* 51 80–92. 10.1016/j.im.2013.09.003

[B78] Wu J. Liu L. Huang L. (2017). Consumer acceptance of mobile payment across time: antecedents and moderating role of diffusion stages. *Indus. Manag. Data Syst.* 117 1761–1776. 10.1108/imds-08-2016-0312

[B79] Wu K. Vassileva J. Zhao Y. Noorian Z. Waldner W. Adaji I. (2016). Complexity or simplicity? Designing product pictures for advertising in online marketplaces. *J. Retail. Consum. Serv.* 28 17–27. 10.1016/j.jretconser.2015.08.009

[B80] Wu L.-H. Wu L.-C. Chang S.-C. (2016). Exploring consumers’ intention to accept smartwatch. *Comput. Hum. Behav.* 64 383–392. 10.1016/j.chb.2016.07.005

[B81] Yang H. Yu J. Zo H. Choi M. (2016). User acceptance of wearable devices: an extended perspective of perceived value. *Telemat. Inform.* 33 256–269. 10.1016/j.tele.2015.08.007

[B82] Yeniyurt S. Townsend J. D. (2003). Does culture explain acceptance of new products in a country: an empirical investigation. *Int. Mark. Rev.* 20 377–396. 10.1108/02651330310485153

[B83] Yokoyama R. Nozawa T. Sugiura M. Yomogida Y. Takeuchi H. Akimoto Y. (2014). The neural bases underlying social risk perception in purchase decisions. *Neuroimage* 91 120–128. 10.1016/j.neuroimage.2014.01.036 24473098

[B84] Yoo B. Donthu N. Lenartowicz T. (2011). Measuring Hofstede’s five dimensions of cultural values at the individual level: development and validation of CVSCALE. *J. Int. Consum. Mark.* 23 193–210.

[B85] Yoo J. Kim M. (2014). The effects of online product presentation on consumer responses: a mental imagery perspective. *J. Bus. Res.* 67 2464–2472. 10.1016/j.jbusres.2014.03.006

[B86] Zhang M. Luo M. Nie R. Zhang Y. (2017). Technical attributes, health attribute, consumer attributes and their roles in adoption intention of healthcare wearable technology. *Int. J. Med. Inform.* 108 97–109. 10.1016/j.ijmedinf.2017.09.016 29132639

